# Experts’ subjective theories: how did they explain post-pandemic school violence in their public discourse through digital media?

**DOI:** 10.3389/fsoc.2026.1731876

**Published:** 2026-03-03

**Authors:** Martina Zelaya, Pablo Castro-Carrasco, Vladimir Caamaño-Vega, Claudia Carrasco-Aguilar, Fabiana Rodríguez-Pastene-Vicencio, Veronica Gubbins, David Cuadra-Martínez, Camila López-Oyarzún

**Affiliations:** 1 Research Center on Subjective Theories, Department of Arts and Letters, Faculty of Humanities, Universidad de La Serena, La Serena, Chile; 2 Research Center on Subjective Theories, Department of Psychology, Faculty of Humanities, Universidad de La Serena, La Serena, Chile; 3 School of Psychology, Universidad Santo Tomás, La Serena, Chile; 4 Department of Didactics and Educational Organization, Universidad de Málaga, Málaga, Spain; 5 Department of Gender, Policy and Culture, Universidad de Playa Ancha, Valparaíso, Chile; 6 Faculty of Education, Psychology and Family, Center for Psychology, Education and Family Research, Universidad Finis Terrae, Santiago, Chile; 7 Department of Psychology, Universidad de Atacama, Copiapó, Chile; 8 Research Center on Subjective Theories, Department of Arts and Letters, Faculty of Humanities, Universidad de La Serena, La Serena, Chile

**Keywords:** COVID-19, digital media communication, expert knowledge, public discourse, school violence, subjective theories

## Abstract

**Introduction:**

In the aftermath of the COVID-19 pandemic, public communications have reported a rise in school violence. This study seeks to understand the collective subjective theories in the public discourse of experts on school violence after educational confinement due to the COVID-19 pandemic. Specifically, it aims at understanding the causes, effects, intervention strategies and contextual conditions associated with the challenges of school violence.

**Methods:**

Drawing on a documentary research design and qualitative methodology, we reconstructed subjective theories based on 109 public discourses on YouTube and Google News, by professionals in education, psychology, and other fields.

**Results:**

Three macro-level subjective theories were identified: Social subjective theory, Educational subjective theory, and Psychological subjective theory. These offer different explanations of the causes of violence, its consequences, and appropriate intervention strategies. Social Subjective Theory emphasizes exclusion, inequality, and systemic abandonment. Psychological Subjective Theory focuses on the deterioration of students’ mental health and emotional distress. Educational Subjective Theory highlights institutional fragmentation and policy contradictions.

**Discussion:**

The findings reveal that expert discourses, besides describing the problem, shape public agendas, justify interventions, and allocate responsibility. The study highlights the public role of expert knowledge in moments of micro and macro-level institutional uncertainty, showing how subjective theories function as interpretive frameworks of educational issues.

## 1 Introduction

Recently, the mass media have reported an increase in school violence following the COVID-19 pandemic. For instance, in December, The New York Times (2024) headlined “Gun Violence at Schools Has Risen Since the Pandemic”, reporting a notable increase in this type of violence in United States schools, and *The Scotsman* newspaper reported a 76% increase in the 110 incidents of school violence over the three years immediately preceding the COVID-19 pandemic (February 2025). School climate has deteriorated globally during and after the COVID-19 pandemic and violence in schools has increased in many countries around the world. In different countries, cyberbullying (Espelage et al., 2024; Molcho et al., 2025), students aggression toward teachers (McMahon et al., 2024), bullying toward students with special educational needs (Cornell et al., 2025), school shootings (Scott et al., 2024), physical and psychological school violence (Imran, 2024), and the influence of structural aspects (Canci et al., 2024) have increased (Forsberg and Thorvaldsen, 2022; Țîroiu, 2023).

This global issue has gained social and political interest, and violence in schools has become a priority focus of international education policies. Such is the case of the 2030 Agenda for Sustainable Development of the United Nations (2015), which has established the need to address the ways of living together in schools in order to foster a culture of collaboration, inclusion, peace, and non-violence, to achieve high-quality education.

After the return to in-person schooling, international studies revealed a complex picture of post-pandemic challenges affecting children and adolescents. Although bullying appeared to decrease during remote learning (Stewart et al., 2024), the reopening of schools coincided with rising levels of violence, anxiety, and school victimization (Anderman et al., 2024; Aylin-Arici et al., 2024; McMahon et al., 2024). In some countries, such as Germany and Bangladesh, increases in youth crime and suicide rates were reported (Nägel and Kroneberg, 2023; Rahman-Himel et al., 2024). Globally, anxiety, depression, and emotional distress have been increasingly rising among children and adolescents after the pandemic (Man Ng and Ling Ng, 2022; Wang et al., 2022). This scenario has posed challenges to various social systems in understanding and addressing these psychological phenomena in specific contexts (Liu et al., 2025). In the field of education, current findings underscore the urgency of designing policies to address the long-term consequences of the pandemic on children and adolescents (Lal and Gupta, 2025).

In the Chilean case, the post-pandemic context has revealed a major crisis in school climate, including violence, insecurity, and an impact on mental health and well-being (Berger, 2024). Thus, a new policy on school climate was recently published, and one of its objectives is to provide a solution to the needs and problems of school climate caused by the COVID-19 pandemic (Castro-Carrasco et al., 2025).

In 2022, the return to in-person schooling was marked by a sharp rise in reports of school violence in Chile, reflecting a broader international concern about the post-pandemic deterioration of students’ mental health and behavior (Rojas-Andrade et al., 2024). The increase in violent incidents and emotional distress among students was not isolated, but part of a global trend documented in recent research (Ashraf et al., 2023; Mazrekaj and De Witte, 2024). This underscores the relevance of analyzing expert discourse during that year, as according to Reveilhac (2022), political authorities and health experts used social media to improve communication with the public during the pandemic.

When dealing with crises, such as a pandemic, it is essential that the stakeholders involved in mitigation, prevention, emergency response and recovery are integrated and work collaboratively. In this context, experts and scholars play a key role in risk management (Rindrasih et al., 2024).

In the case of the education emergency caused by the COVID-19 pandemic, the impact on the educational process among 94% of students globally included interruption of teaching, damage to the psychological health of the educational community, poor quality of learning, problems in social–emotional development, deterioration of school climate and an increase in violence (Durrani and Ozawa, 2024). This impact could, in part, be mitigated with appropriate risk management. Tipler et al. (2018) have proposed that adequate risk management in education requires schools to prepare for crises and to receive expert professional support from external institutions.

Accordingly, experts need to show professionalism in the face of emergencies, expressed in the ability to adapt their discourse and services in accordance with emerging social demands (Ray, 2024). Precisely, experts had an important presence in the media during and after the COVID-19 pandemic, guiding the response to physical and psychological health risk, the economic damage caused by the pandemic, education in emergencies and the functioning of society in general (Rouillard et al., 2023).

Experts are often recognized as such due to their specialized knowledge, skills, or their institutional or professional roles (Flick, 2022). However, expert knowledge is not limited to formalized, academic understanding, it also encompasses practical, experience-based knowledge that may not always be explicitly articulated. The relevance of studying expert knowledge is that it can become dominant within a field, shaping the practices and decisions of others (Bogner et al., 2018).

Along with the aforementioned, a relevant dimension of people in the pandemic context was the subjective knowledge that was constructed and shared by different means to respond to the pandemic, for example, beliefs about protective measures (Escouflaire et al., 2024). In the case of professionals, according to Polat et al. (2024), the pandemic context challenged their agency and professional identity, having to face uncertainty based on their experience—professional and subjective—to deliver the services that society demanded in and after the emergency. Additionally, online media gained prominence in the face of containment measures (Reveilhac, 2022), with social media facilitating public health communication and pandemic management through expert engagement (Abbas et al., 2021). This, faced with the significant challenge of conveying factual information and countering conspiracy beliefs that circulated about the pandemic and mitigation measures (Nugent et al., 2025).

Based on the above, this study is aimed at understanding experts’ discourse on school violence on publicly accessible digital platforms during the year 2022—after the pandemic. This study is particularly interested in the experts’ argumentative and explanatory beliefs on these issues, i.e., their subjective theories (Groeben and Scheele, 2020). This is because subjective theories are a type of belief that allow people to justify, explain and direct their actions, as well as to understand the world in general (Flick, 2023). In psychosocial studies of professionals, there is significant scientific literature that shows that these actors use subjective theories to make professional decisions and guide their professional practice (Castro-Carrasco et al., 2024; Goller and Markert, 2024), especially when they do not have objective knowledge (Jancic, 2015) or the work context requires novel and urgent responses.

The main objective of this study is to understand the collective subjective theories in the public discourse of experts on school climate and school violence after educational confinement. Specifically, it aims at understanding the causes, effects, intervention strategies and contextual conditions associated with the challenges of school climate and school violence. This study seeks to answer the following research questions:

1. Based on public discourse by experts in Chile in 2022, how do experts’ subjective theories on school violence after lockdown converge or diverge?
2. In these theories, what causes certain attributions of responsibility to be emphasized or minimized (families, school/teachers, students, state/justice, society) when explaining the phenomenon?
3. Do these theories justify certain intervention strategies and, at the same time, leave out others, in relation to the causes, effects, and contextual conditions of school violence?

### 1.1 School violence and COVID-19

In Chile and South America, school violence has intensified and become a “public problem” (Varela et al., 2024), as well as in other places in the world. School violence has increased in different countries (Forsberg and Thorvaldsen, 2022), with cases such as cyberbullying (Espelage et al., 2024; Molcho et al., 2025), aggressions from students toward teachers (Reddy et al., 2024), bullying against students with special educational needs (Cornell et al., 2025), shootings (Scott et al., 2024), physical and psychological school violence (Imran, 2024), and the influence of systemic factors (Canci et al., 2024). In this research, school violence is understood in a non-reductionist and ecological perspective (Toledo et al., 2018). It is not an individual issue and the context is what matters (Castro-Carrasco et al., 2024), along with the collective and subjective perspective of experts. School violence is linked to pedagogical shortcomings and a lack of socioemotional training, making it necessary to implement comprehensive strategies based on dialog, values, and prevention involving the entire educational community (Alonso-Rodríguez et al., 2025; Min et al., 2024; Santamaría-Villar et al., 2021).

School violence is currently understood as a post-pandemic problem at the global level and, therefore, an alarming challenge for schools, especially those with fewer resources (Mosito and Sitoyi, 2024). Although there is no single definition on school violence, it can be understood as deliberate acts that result in harm to people in the educational community (Karakuş, 2022). It involves violent and aggressive actions committed in school context, such as physical aggression and cyberbullying. Also, disruptive behaviors such as pushing, fighting, hitting and throwing objects that are intended to cause harm, in addition to hostility through digital media and bullying (Freeman et al., 2024). It is known that students who face or witness violent episodes experience negative repercussions in their educational performance, with lower grades compared to those who are not exposed to violence (González-Moreno and Molero-Jurado, 2023).

These violent behaviors are rooted in a network of internal and external influences from the school context, individual (e.g., aggression and low social–emotional skills), family (e.g., dysfunctionality and violence), community, social and political factors (Cunha et al., 2024). Therefore, a possible explanation of school violence is the manifestation of structural dynamics of exclusion, injustice, and marginalization that permeate social life and are reproduced within educational spaces. From this perspective, aggression, harassment, and conflict in schools reflect social, economic, cultural, or gender-based inequalities that place certain groups of students in positions of vulnerability and stigmatization (Liu et al., 2025; Pabayo et al., 2022; Qorbani et al., 2022).

In the pandemic and post-pandemic context, school climate presented a significant deterioration, and school violence had an alarming increase (Țîroiu, 2023). Education suffered a deep transformation due to the pandemic impact (Vallejos-Parás and Covarrubias Díaz-Couder, 2024). The ways of living together in educational institutions were altered and interrupted, significantly affecting the socioemotional development of students, value formation, citizenship education, mental health and the well-being of educational communities (Córdoba, 2024). The work of Sandner et al. (2023) showed that the closure of educational institutions, as a result of COVID-19, had an immediate positive effect on the well-being of students. However, it strongly decreased during and after the pandemic, along with the deterioration of mental health, which was associated with the modifications of school routines, such as educational programs (Faúndez et al., 2025). Faced with this, education experts in Chile sought to mitigate the effects of the pandemic on school climate and school violence by using the media for this purpose.

### 1.2 The role of experts during the pandemic and the mass media

While there is no full consensus on what it means to be an expert, it can be argued that it corresponds to someone who has in-depth knowledge on a topic and/or the ability to perform a task with a specialized and recognized mastery, which is achieved with experience and training in the area (Costello and Rutherford, 2019). From a sociological perspective, experts can also be understood as parts of particular social groups that occupy influential positions in public discourse, often being described as influential and prominent. In modern societies, experts are frequently constructed as agents of truth and authority whose voices are expected to guide public understanding and political decision-making (Bogner et al., 2018). However, rather than approaching expert knowledge as a fixed or canonical body of objective facts, this study understands expertise as a form of socially situated knowledge. From this standpoint, experts are relevant because of the ways in which their interpretations and explanations shape social practices and public agendas. That is, the ways in which their explanations influence public understanding when disseminated by the media.

According to Lavazza and Farina (2020), an expert becomes trustworthy when they have an adequate academic background, experience, achievements and reputation in their field, are politically impartial and assume their performance with ethical commitment and motivation. According to the same author, although people’s trust in scientific-technical knowledge has decreased in recent times, during the COVID-19 pandemic the population heeded the advice of experts, and politicians entrusted them with much of the responsibility for dealing with the pandemic.

Similarly, Rouillard et al. (2023) point out that during the COVID-19 pandemic, experts played an important role in addressing the crisis and post-disaster reconstruction. This role was played mainly through social media, which enabled communication with the public, given the restrictive measures adopted at the time (Abbas et al., 2021; Reveilhac, 2022). This can be seen under the scope of relevant research in the area that shows that the mass media can distort the public perception of school violence, for instance, regarding its proliferation even when scientific evidence shows that there was no significant increase of school violence (Lawrence and Mueller, 2003).

According to Anwar et al. (2020), the media enabled experts and politicians to spread pandemic protection measures globally. In the context of the pandemic, expert discourse was expected to be reliable, capable of counteracting the misinformation that often circulated on social media (Haupt and Parmet, 2022). Although a more detailed analysis revealed that trust in experts and health authorities was high at the beginning of the pandemic, in the course of the pandemic it weakened, leading to the population also turning to non-scientific information on how to protect themselves from the effects of the pandemic (Battiston et al., 2021; Reveilhac, 2022).

In line with their public role during the pandemic, experts did not only provide technical knowledge but also expressed a heterogeneous set of subjective orientations, rules, normative stances, and personal interpretations about reality, known as interpretative knowledge (Bogner et al., 2018). This type of knowledge is not necessarily more accurate than that of others, but it reflects the expert’s particular perspective on the issue, which are gaining relevance in public discourse and their potential influence on public policy (Sorial, 2017).

Regarding school climate and school violence after the pandemic, the Chilean Teachers’ Union was responsible for broadcasting a large amount of news on the web. Castro-Carrasco et al. (2024) reviewed these news items and found subjective theories about the meanings attributed to school violence and school climate, in addition to a set of measures aimed at promoting inclusive policies, curricular adjustments, increased funding for public education to improve mental health and working conditions, which are viewed as key elements in the management of positive school climates.

### 1.3 Professionals’ subjective theories

The study of subjective theories originates in German psychological research and is historically rooted in the psychology of the reflexive subject (Groeben and Scheele, 2000), which draws on the personal constructs theory (Kelly, 1991), thus comparing scientific theorization and the everyday explanations people construct to make sense of their lives (Catalán, 2016). More broadly, subjective theories research is aligned with a constructivist approach and has often been associated with the constructivist paradigm. It is assumed that subjective theories are explanatory models constructed in interaction with the environment, whose objective is to explain some dimension of the social world based on a sociocultural context (Sandoval and Cuadra, 2018).

Subjective theories are a type of belief that have an argumentative structure that allows describing, explaining, justifying and guiding people’s actions (Flick, 2023). In professional training and practice these have an important role, since they allow professional decisions to be made when scientific knowledge is lacking, or it is necessary to act upon urgent and unforeseen situations (Jancic, 2015).

Professional Subjective Theories (Cuadra et al., 2018), refer to professionals’ subjective frameworks that enable them to interpret, anticipate, and address complex problems in dynamic contexts, particularly when technical or scientific knowledge proves insufficient. This contrasts with Flick’s (2022) definition, which restricts subjective theories to the non-professional or lay knowledge held by professionals.

Subjective theories do not always succeed in integrating scientific knowledge and may even play a leading role in explaining phenomena and behaviors, in addition to guiding professional action. Often, specially planned formative strategies are required to achieve the integration of these intuitive explanations with scientific knowledge (Cuadra et al., 2018). On this, and in the case of teachers, several decades ago Dann et al. (1987) demonstrated that an important difference between successful and unsuccessful teachers on the management of aggression in the classroom, is that the former have more complex subjective theories integrated with scientific knowledge, so that the causes of violence can be identified, and teaching decisions can be made.

Previous research has drawn on subjective theories to explore teachers’ perspectives on issues such as education for sustainability (Goller and Markert, 2024; Lohmann and Goller, 2023) and educational quality (Niyibizi, 2024). The contribution of nurses’ subjective theories or representations to the study of nursing care has also been argued (Flick et al., 2002; Garay Rivera and Garay Rivera, 2025). Hence, studying the subjective theories of experts on school violence becomes especially relevant, given that the obtained results not only allow us to complement the psychosocial theory of professionals, but also to guide the initial and continuing professional training of experts in education.

## 2 Methods

### 2.1 Type of study, methodology, and design

This study followed a documentary research design (Bowen, 2009) using qualitative methodology with a descriptive and interpretive approach. It was grounded in a comparative case study design (Creswell and Creswell, 2020; Flick, 2023) aimed at analyzing the public discourse of experts on school violence in Chile during 2022, disseminated through digital platforms. The study focused on the professional subjective theories (Cuadra et al., 2018; Flick, 1989, 2023; Garay Rivera and Garay Rivera, 2025) expressed by professionals publicly recognized as experts in areas such as psychology, education, sociology, medicine, and social work. This perspective draws on discursive psychology (Camic, 2021), as subjective theories constitute a psychological approach to discourse analysis.

The cases under study were constituted by a corpus of 109 expert discourses—68 obtained from YouTube and 41 from Google News—produced in the aftermath of the return to in-person schooling following the COVID-19 lockdown. YouTube was selected as a platform for data collection due to its widespread use and accessibility in Chile. By 2022, it had reached 81.4% of the national population, making it one of the most influential digital spaces for public communication (Branch, 2022). As the largest open-access audiovisual archive in the world, YouTube enables users to upload and share long-form videos without temporal or geographic restrictions, facilitating the dissemination of discourses to broad audiences (López, 2018). Google News was included as a data source given Google’s dominant role as the leading search engine worldwide (Błajda et al., 2024).

Given that this study uses media discourse as a documentary source, it is important to note that the media filters information and sources through a combination of traditional editorial processes and, more recently, personalization algorithms (Kitchens et al., 2020), while also taking social dynamics into account. In traditional media, journalists and editors select what to publish based on criteria such as relevance, novelty, public interest, and editorial line. This gatekeeping process (White, 1950) prioritizes certain topics and expert sources and may be influenced by economic, political, or ideological interests (Corrêa da Silva, 2022). In digital environments, platforms use algorithms that filter and prioritize news, which can create “filter bubbles” and limit exposure to diverse perspectives (Dahlgren, 2021). Additionally, there are commercial and ownership criteria (profit orientation and dependence on advertising) and virality on social media, where users’ selection and sharing can amplify sensationalist or polarizing content, regardless of its veracity (Rhodes, 2022). Together, these mechanisms affect both the diversity and quality of the information that reaches the public, potentially reinforcing biases and promoting polarization (Corrêa da Silva, 2022).

In this study public discourse is understood as interactions that take place through a broadcast platform, either spoken or written, in which the discourse is oriented to a reader, listener or viewer who is not physically present (Tavadze et al., 2024). As such, discourses articulate social patterns such as domination, discrimination, ideologies and the idea of common sense (Catalano and Waugh, 2020).

The main objective of this study is to understand the collective subjective theories in the public discourse of experts on school violence after educational confinement. Specifically, it aims at understanding the causes, effects, intervention strategies and contextual conditions associated with the challenges of school violence.

### 2.2 Inclusion and exclusion criteria

The following criteria were established to determine the eligibility of materials for analysis:

#### 2.2.1 Inclusion criteria

- Content must address or be semantically related to the phenomenon of school violence in Chile during 2022.
- The materials—either written texts or interviews in video/audio format—must have been broadcast through publicly accessible digital platforms in 2022.
- The selected materials must feature individuals recognized as experts, specialists, or professionals, whose perspectives were broadcast by media outlets, institutions, or individuals that framed their discourse as relevant.
- The materials must have been distributed by regional or national media, institutions, or public figures.

#### 2.2.2 Exclusion criteria

- Content not semantically related to school violence in Chile following the return to in-person education.
- Materials published after 2022, even if they addressed post-pandemic school violence.
- Perspectives presented in media shows or news segments by individuals who were not explicitly considered as experts in the field.
- Materials of unverifiable authenticity.
- Discourse by individuals affiliated with the research team or those who had co-authored publications with any team member in the past 5 years.

### 2.3 Sampling

In this study, the categories included experts in psychology and experts from other professional domains (e.g., education, medicine, sociology, social work). Two researchers allocated a total of 20 h to perform the initial sampling, which involved searching for expert discourse in YouTube videos and Google News. The search focused on both national and regional media appearances.

The final corpus consisted of 109 expert discourses: 68 from YouTube and 41 from Google News.

The sampling strategy was guided by the concept of sample structure beforehand (Flick, 2023), with inclusion and exclusion criteria prior to data collection. Additionally, the adequacy of the final sample was evaluated based on the principle of information power (Camic, 2021). According to this framework, the strength and relevance of the sample depend on five key dimensions: the study aim, sample specificity, use of established theory, quality of dialog, and analysis strategy. Given the above-mentioned, the sample size of 109 expert discourses was deemed sufficient.

### 2.4 Data analysis

This study followed a coding process that adapts conventional coding (Creswell and Creswell, 2020) by creating one or more emerging codes from the discursive units (videos or web news). Thus, the analytical process followed three main stages. The subjective theories are hierarchically located in a belief system. In this study, a distinction was made between three types of subjective theories according to their hierarchy (Catalán, 2016), application domain (more general or specific topics to which they are applied) (Kelly, 1991), and quantity, from the most general (usually few) to the most particular or restricted.

Initially, a within-case analysis was conducted in which micro-level subjective theories were reconstructed. These theories were formulated as explanatory conditional statements, such as “If X, then Y” (Dann, 1989). A total of 109 micro-level theories were identified. During the process, six deviant cases (Flick, 2023) were excluded from the analysis. These cases exhibited thematic and conceptual divergence from the emergent categories and did not fit within the overarching patterns identified in the rest of the corpus.

In the second stage, a cross-case analysis (Miles et al., 2019) grouped the resulting 103 micro-level subjective theories into 12 intermediate-level subjective theories based on thematic similarities and shared assumptions. Finally, a relational analysis (Creswell and Creswell, 2020) synthesized the intermediate-level subjective theories into three macro-level subjective theories, representing distinct epistemological and disciplinary orientations.

- Educational Subjective Theory: Explanations grounded in teaching and institutional dynamics.
- Psychological Subjective Theory: Explanations based on individual and mental health factors.
- Social Subjective Theory: Explanations that emphasized structural, cultural, and socioeconomic determinants.

In order to illustrate the coding trajectory followed in this study, Table 1 presents exemplary cases of the analytical process from raw data to higher-order abstraction. The Table shows selected excerpts from the corpus (raw quotes), and their successive transformation into micro-level subjective theories, intermediate (meso-level) subjective theories, and macro-level subjective theories. One illustrative example is provided for each macro-level subjective theory (Educational, Psychological, and Social), allowing the reader to trace how expert discourse was progressively interpreted across the different stages of analysis.

**Table 1:** Coding trajectory from raw data to macro-level subjective theories.

Raw data (quotes)	Micro-level subjective theory	Meso-level subjective theory	Macro-level subjective theory
“*We have been experiencing violence for years, perhaps decades, and it has been escalating. Violent patterns have been reproduced, violent interactions have been reproduced by young people, fathers, mothers, and that, added to the pandemic, has created a kind of pressure cooker.*” (Psychologist 86)	If we have been exposed to violent models and interactions for years, then we need to take a critical look at how to prevent violence in schools.	Since violent patterns of society are reproduced in schools, then conflicts are likely to occur (Society).	If behavior depends on the environment, then the school violence events reflect what happens in society (Social subjective theory).
“*Schools are aware of the violent incidents that occur daily in classrooms and during recess. However, identifying cases of bullying becomes more complex due to the characteristics that must be present in order to classify it as such. This is why each school has protocols for dealing with bullying, which include identifying the appropriate professionals to handle such cases.*” (Special needs teacher 77)	Given that it is not easy to identify cases of bullying, it is necessary for each school to have action protocols in place that designate who are the appropriate professionals to identify and address it.	If we want to deal with SV, then we must update the protocols and combine them with formative strategies that promote good treatment (Protocols and policies).	If we want to have a good school climate, then we must focus on formative practices that build a positive school climate within the educational community (Educational subjective theory).
“*The return to school has been challenging for everyone [.] In schools, it was impossible to expect a return to a full school day. That is, after two years without classes, without school, without sharing, suddenly we have to spend ten hours a day, wearing masks, in very small spaces [.] This is a generation that does not know how to solve conflicts. They are socio-emotionally illiterate.*” (Psychologist 64)	Given that returning to school has been challenging for everyone, it is necessary to seek actions and strategies that relieve the socio-emotional and psychological effects on students.	Since SV is related to the worsening of mental health, then the psychological and emotional struggles of students need to be addressed (Mental health).	If there is inconsistency between mental health care and educational standards, then school violence and psychological difficulties are to be expected (Psychological subjective theory).

### 2.5 Scientific rigor criteria

To ensure scientific rigor, this study prioritized transparency, reflexivity and intersubjectivity (Catalán, 2021; Vasilachis de Gialdino, 2019). Transparency was addressed by providing the reader with detailed information on how the data were obtained, selected, and analyzed. Reflexivity was maintained through the researchers’ ongoing examination of their own interpretive choices while reconstructing and categorizing the subjective theories. Finally, intersubjectivity was reached through collaborative analysis and discussion among the research team, which allowed for the refinement of the results based on shared criteria.

## 3 Results

As stated previously, four subjective theories were excluded from the cross-case analysis due to overlapping features between the social and educational, and the social and psychological domains. Additionally, two subjective theories were excluded as they reflected a more judicial perspective; these were categorized during the within-case analysis as “Judicial Subjective Theory.” Given that these two share distinct features with the three main subjective theories, they will be presented below.

*ST 100*: Since the system and authorities do not respond to complaints, individuals resort to acts of public exposure (from Spanish, *funas*) on social media to bring visibility to these cases.

*ST 81*: Given that cyberbullying takes place in a context of virtual interaction, we need to understand that this gives the attackers “almost absolute anonymity”, which makes it harder to access evidence and proof.

These two Judicial Subjective Theories reflect a distinct explanatory logic that frames school violence through a lens of legal accountability, institutional failure, and the need for alternative forms of justice and access to evidence.

Regarding the cross-case analysis, Table 2 below shows the 12 intermediate level subjective theories. These intermediate subjective theories represent recurring explanatory patterns articulated by professionals across different fields, such as education, psychology, and social work, regarding the post-pandemic challenges of school violence in Chile. Each intermediate subjective theory synthesizes a set of similar micro-level explanations, highlighting shared beliefs about causes, effects, and interventions.

**Table 2:** Intermediate level subjective theories.

Reconstructed subjective theory	Main themes associated
If we want to address cyberbullying, then we must educate in values and principles in classrooms	Cyberbullying
If we want to improve the SV problem, then teachers must have social–emotional skills and promote well-being.	Teachers’ skills
If we want to reduce vandalism and delinquency, then we must establish formative measures developed within the educational community.	Criminalization, judicial approach to school violence
If we want to achieve good SC, then we must address the formative aspects (of education) in safe spaces.	Formative model
If families educate in values and create spaces for bonding with their children, then the problem of violence can be addressed.	Family and school
If we want to deal with SV, then we must update the protocols and combine them with formative strategies that promote good treatment.	Protocols and policies
Since SV is related to the worsening of mental health, then the psychological and emotional struggles of students need to be addressed.	Mental health
Since educational contexts are culturally diverse, it is essential to integrate SC with anti-racism and interculturalism in dignified spaces.	Cultural diversity
If we want to address SV, then we must understand the reality of each school and dialog with both the educational community and authorities.	Dialog
Since the pandemic affected the development of social–emotional skills, it is essential to foster students’ holistic development and strengthen bonds.	Social–emotional development and the Pandemic
If we want to prevent conflicts and cases of SV, then we must teach values and good treatment from an early age.	Preventing school violence and conflict
Since violent patterns of society are reproduced in schools, then conflicts are likely to occur.	Society

### 3.1 Macro-level subjective theories

Building upon the thematic patterns identified at the intermediate level, the following section presents the three macro-level subjective theories reconstructed from the expert discourses. These broader theories condense the underlying assumptions and explanatory logics shared across various experts’ perspectives. Each macro-level subjective theory reflects a distinct disciplinary orientation (educational, psychological, or social) through which experts made sense of the deterioration of school climate and the rise of school violence in the aftermath of the pandemic.

#### 3.1.1 Educational subjective theory

*Main subjective theory*: If we want to have a good school climate, then we must focus on formative practices that build a positive school climate within the educational community.

The Educational Subjective Theory emphasizes that a positive climate is not simply the absence of conflict but the outcome of pedagogical efforts aimed at developing social–emotional skills, values, and participation (ST 5, ST 9). Experts highlighted the relevance of creating safe and formative spaces that nurture empathy (ST 35, ST 72). These spaces are framed as essential for fostering prosocial behaviors and preventing school violence, including bullying and cyberbullying (ST 39). Rather than relying on punitive approaches, experts advocate for the fostering of a shared culture of good treatment, respect, and climate that permeates daily school interactions (ST 10, ST 107).

A recurring theme in the discourses is the need to strengthen the role of educators and families as agents of formative intervention. Experts argue that socioemotional training for teachers, along with their ability to model non-violent conflict resolution, can positively influence students’ behavior and learning. This notion is extended to families, which are seen not only as responsible for instilling values and limits but also as participants in building school interactions (ST 11, ST 19, ST 85). Along with this, protocols alone are deemed as not sufficient, as their effectiveness depends on being implemented within a broader ecosystem of trust, empathy, and shared responsibility, according to the experts (ST 7, ST 18).

Another idea is that formative practices must be adapted to the specific realities and needs of each school (ST 16). Experts express concern that too generic interventions may fail to address the complexity of school life, particularly after the pandemic, as emotional vulnerabilities and digital violence have intensified (ST 56, ST 59). These discourses emphasize the relevance of contextualizing actions (ST 48), promoting dialog with authorities, and designing interventions collaboratively (ST 57).

According to expert discourse, education must be reoriented toward emotional and relational dimensions (ST 7, ST 9). This implies incorporating strategies that foster empathy and digital awareness (ST 80). The idea of “learning to coexist” involves creating plans that include the entire educational community, with the support of public policies (ST 44).

As an expert quotes, “The school must take responsibility for its prevention protocols and disciplinary measures […] lasting measures must be put in place so that other students who are watching do not follow suit and it does not become a trend.”. Based on this quote, we can reconstruct their argument as follows: If we want to reduce bullying, then we must raise awareness of the role that protocols and disciplinary measures fulfill in schools (ST94). In this subjective theory, school violence is understood as a phenomenon that must be addressed through institutional responsibility and pedagogical regulation. The expert frames prevention as a collective and formative task of the school, where protocols and disciplinary measures function as pedagogical signals that shape students’ behavior. From their perspective, disciplinary actions are formative as they communicate norms and boundaries to the entire educational community, explicitly including those who witness violent acts.

#### 3.1.2 Psychological subjective theory

*Main subjective theory*: If there is inconsistency between mental health care and educational standards, then school violence and psychological difficulties are to be expected.

The Psychological Subjective Theory frames school violence as a symptom (ST 38) of unresolved emotional distress and deteriorating mental health, particularly after the pandemic (ST 62). Thus, experts call for a response in which mental health support is integrated into the functioning of schools, alongside instruction (ST 20, ST 24). For example, a quote that supports ST 62 is.

“*It has not been easy for educational communities to return to physical attendance and the usual rhythm of operation in their schools. There have been various situations of aggression, threats, fights, harassment, and physical and sexual abuse among students and also towards/among teachers, which have been experienced in schools since the beginning of the school year. All of these are manifestations of physical, verbal, and sexual violence, which arise and are expressed as a clear sign that something is not right and that we must urgently address* […] *the importance of the socio-emotional well-being of students and teachers as key elements for the deployment of any educational process. Taking the time to find out how the members of an educational community are doing, what their experiences, emotions, and feelings are, as an essential milestone in learning.*” (Social worker).

We can reconstruct this expert’s argument as follows: If an educational community returns to face-to-face learning with aggression and conflict, then this is a sign of distress; therefore, if the social–emotional well-being of students and teachers is not prioritized, then learning weakens (ST 62). In this subjective theory, the educational community is conceived as an “organism” that can experience, metaphorically, psychological distress, identifiable through observable signs (aggression, threats, fights, harassment, and abuse). Consequently, the solution to school violence is formulated as the need to remove these signs through actions focused on the social–emotional well-being and mental health of students and teachers. Although this explanation highlights a relevant issue, it tends to psychologize the phenomenon and reduce its understanding and approach to a deterioration of the “emotional state” of the community. Furthermore, it does not incorporate a situated understanding of school violence as a relational and interactive problem between groups and/or individuals in a specific educational context. Considering that school violence is a social phenomenon, there are limitations in the explanatory and interventionist scope of this formulation.

A tenet of this theory is the need to address the emotional challenges associated with the return to in-person schooling. Experts highlight that depression, anxiety, and emotional dysregulation became more prominent during the pandemic, and that these conditions escalated into disruptive and violent behaviors in schools (ST 13). Interventions, therefore, must not be limited to reacting after violence occurs; instead, experts advocate for preventive and reparative strategies that begin before students start their classes and continue after conflict takes place (ST 22, ST 26). These include psychological evaluations, socioemotional learning programs, routine restructuring, and development of empathy and communication skills (ST 17, ST 31).

Experts discourses also stress the relevance of working alongside students, teachers, families and caregivers to create a network of emotional support (ST 93). Experts argue that prevention of violence requires equipping teachers with tools to recognize early signs of emotional distress and respond appropriately (ST 38).

#### 3.1.3 Social subjective theory

*Main subjective theory*: If behavior depends on the environment, then the school violence events reflect what happens in society.

The Social Subjective Theory conceptualizes school violence as a mirror of broader societal dynamics, and experts argue that students’ behavior in educational settings are deeply shaped by their social environments (ST 53). According to experts, the roots of violence are not confined to the individual or the school itself but are embedded in larger systemic structures marked by inequality (ST 88), social unrest (ST 65), and cultural normalization of aggression (ST 89, ST 108).

These explanations can be seen in the following quote: “*The social unrest brought the dirt under the carpet into the open, and now we can see situations of injustice more clearly. Children and adolescents are very sensitive to issues of social justice* […] *The two years of the pandemic have caused great hardship for students*”. We can reconstruct the experts’ argument as follows: If we want children and adolescents to understand the rules of coexistence in schools, we must address their sensitivity to social injustice, as made visible by the social unrest and the pandemic (ST 65). In this subjective theory, school violence is interpreted as a manifestation of broader social dynamics that exceed the boundaries of the school as an institution. This expert frames students’ violent behaviors as a response to structural conditions such as injustice and collective disruption. Following this argument, schools are not seen as spaces capable of fully regulating students due to societal tensions and violent modes of interaction that are being reproduced. While this subjective theory offers an explanation that situates school violence within wider processes of social reproduction, it tends to externalize responsibility from the schools, potentially limiting the scope of school-based agency and intervention in addressing violence within everyday educational practices.

Experts frame school violence as a phenomenon that reflects prevailing modes of interaction in families, communities and media (ST 27, ST 49), including the rise of hostile digital environments, as in ST 92: Since children and young people were more exposed to violent content on social media during the pandemic, we must understand that this has led to “violent behaviors that are validated by the digital environment”.

Some experts point out that children and adolescents often replicate behaviors learned in families in which violence is used as a means of conflict resolution (ST 42) or where parental presence and emotional availability are limited (ST 43). The normalization of such interactions, experts say, fosters a culture where aggression becomes a tool for asserting identity or gaining visibility, especially among youth who feel abandoned or ignored (ST 90). Thus, violence is interpreted as invisibility and as a strategy for recognition, shaped by the way media and society portray power and attention (ST 27).

Finally, experts warn that current limitations in citizenship education and the interruption of peer relationships during the pandemic have undermined students’ abilities to reengage respectfully in collective life (ST 66, ST 70). In order to reduce school violence, there must be “dignified, protective, and plural educational spaces” (ST 60) where children and youth can recognize diversity as a constitutive feature of social life (ST 4).

### 3.2 Relational results

**Figure 1:**
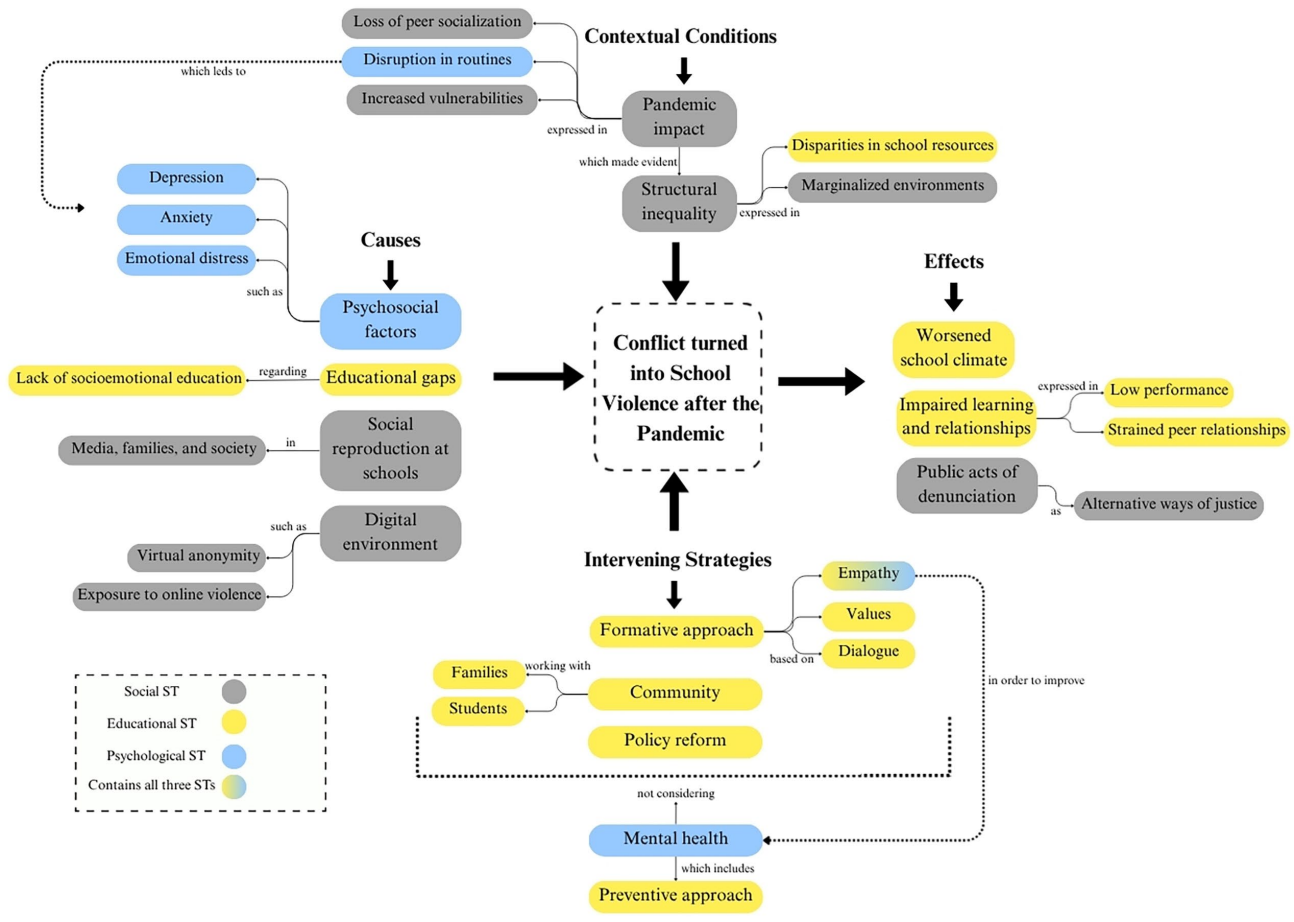
Relational model between 3 macro-level subjective theories.

The results from the final relational analysis are shown in Figure 1.

The three macro-level subjective theories together make a network of explanations that collectively show how school violence emerged and intensified after the pandemic. The Educational Subjective Theory focuses on formative gaps within schools, pointing to a lack of socioemotional learning, and value-based education as a strategy to solve this issue. The Psychological Subjective Theory, in turn, frames school violence as a manifestation of unsolved emotional struggles, such as anxiety and depression, exacerbated by the loss of routines and peer bonds during the pandemic. Finally, the Social Subjective Theory operates at a more structural level, positioning school violence as a reflection of broader societal inequalities, marginalization and the reproduction of violent norms across families, media and digital environments. This macro-level subjective theory establishes the contextual backdrop against which both Educational and Psychological Subjective Theories unfold.

## 4 Discussion

This study sought to reconstruct and analyze the subjective theories expressed by publicly recognized experts in Chile regarding school violence in 2022. Just as scientific literature distinguishes between explanations derived from individual, school, or non-school approaches (Puschmann, 2024) our findings show that these subjective theories are articulated around three dominant explanatory frameworks: educational, psychological, and social. According to our analytical frame, each of these foregrounds different causes, consequences and strategies of intervention. These frameworks reflect discursive positions that shape how school violence is understood, justified and addressed in the public sphere through the media communications, just as previous studies that have shown the relation between the configurations made by the media about school violence and the decisions that are made by policy-makers (Herda-Rapp, 2003).

The Educational Subjective Theory focuses on pedagogical breakdowns and the weakening of school-based formation as causes of school violence. From this lens, the erosion of socioemotional education, the rigid or outdated implementation of coexistence protocols, and the insufficient training of teachers are seen as contributors to the post-pandemic crisis in school climate. This resonates with international research highlighting the role of schools in the development of socioemotional skills after the pandemic (Min et al., 2024), and conflict resolution skills (Santamaría-Villar et al., 2021). Although this explanation focuses on the development of psychosocial skills, its framework is educational rather than solely psychological, thereby considering the development of these skills as a curricular issue and, therefore, a matter for planning and addressing school violence within schools., experts adopting this subjective theory call for formative strategies that foster dialog and promote values, aligning with evidence in research demonstrating greater effectiveness in interventions focused on education and centered on prevention and promotion, such as whole-school approaches to violence prevention (Alonso-Rodríguez et al., 2025; Rojas-Andrade et al., 2024).

The Psychological Subjective Theory, by contrast, situates school violence within a framework of emotional vulnerability and mental health deterioration among students. Experts holding this subjective theory emphasize the effects of prolonged isolation and dysregulation caused by the pandemic, demanding a stronger integration of psychosocial support within the school system. This explanation finds support in recent studies that document rising levels of anxiety, depression, and emotional distress among children and adolescents after the pandemic (Man Ng and Ling Ng, 2022; Wang et al., 2022). Thus, the Psychological Subjective Theory understands school violence as an expression of unprocessed suffering requiring individualized and preventive care, which is also part of the Educational Subjective Theory. Summarizing, this subjective theory aligns with the individual approach (Puschmann, 2024), as referred to in previous research.

In this sense, this type of explanation seems to blur the role of the school when addressing school violence and its influence as the context in which it occurs. As such, it could be functioning as an argument that inhibits the school’s actions or agency. It is an argument that externalizes the school problem to a psychological and health context. This is consistent with the common finding that teachers externalize the causes and responsibilities for school problems, usually considering them to be internal to the student and therefore outside the scope of education (Carroll et al., 2023; González González et al., 2019).

Thus, the finding of the Psychological Subjective Theory does not imply that intervention within the school is impossible, but rather that the work that needs to be done is mainly on a psychological level. The expert argument presented in this subjective theory matches some of the explanations given by another relevant educational actor, such as the Chilean Ministry of Education, in the first year of the return to in-person classes. This institution has emphasized health factors and psychological aspects, such as the deficit of emotional self-regulation, both as causes of school violence issues and as a policy to be implemented, thus leading to a psychologization of the problem (Castro-Carrasco et al., 2025; Ministerio de Educación, 2024; Vargas-Pérez et al., 2024). This psychologization of the problem could lead to reductionist approaches to intervention. However, such interventions should not be dismissed, as some show greater support in the area of school climate. Specifically, Tapullima-Mori et al. (2024), in a systematic review of interventions, highlight that those based on the development of emotional intelligence are among the most effective, although this finding could be influenced by the prevalence of this type of approach in interventions that are commonly implemented precisely because of the significant incidence of this way of understanding the problem. Another interesting finding is that this Psychological Subjective Theory is not mainly represented in this study by experts in the field of mental health. Rather, expert psychologists within the educational context tend to offer a psychoeducational explanation over the individual psychological argument.

The Social Subjective Theory, finally, frames school violence as a symptom of broader societal fractures. Experts drawing on this perspective point to systemic inequality, societal patterns of aggression, and the digital environment as underlying conditions that normalize violent behavior. This aligns with educational and sociological research that conceptualize school violence as an expression of dynamics of exclusion, injustice, and marginalization (Pabayo et al., 2022; Qorbani et al., 2022). From a critical point of view, this subjective theory is also externalizing the problem, as the Psychological Subjective Theory, and negatively implies focusing on aspects that are probably difficult to change within schools, once again taking away the agency from this institution. However, if one expands the view on schooling to include a political dimension, this subjective theory aligns with perspectives in which resistance and the role of schools in social change are relevant (Giroux, 2024). This explanation associated with school violence has also been found when studying the subjective theories of the Chilean Teachers’ Union (Castro-Carrasco et al., 2024).

The judicializing subjective theories identified in this study, although less prevalent, reveal growing concerns with legal framing of school violence. These subjective theories suggest a discursive shift toward punitive approaches, which contrasts with formative and damage-repairing orientation of most experts. The tension between punitive versus formative approaches reflects debates in the literature about the risks of this approach when dealing with conflict at school (Ortiz-Mallegas et al., 2023). On the other hand, Latin American (Adduci, 2020; Mota and Gois, 2023) and worldwide research have revealed a concerning tendency in the media to address school violence from a criminalizing and delictual perspective associated with reactionary responses (Mosqueda et al., 2021). Nonetheless, our findings show that this approach is less prevalent in Chile, among experts consulted by the media. This may be positive, given that specialists in the field are interested in developing a social construct of school violence that does not stigmatize or individualize the phenomenon from a criminal perspective (di Napoli and Sáez, 2022). As such, public discourse would influence the design of comprehensive educational policies.

Expert discourse disseminated through digital media is inevitably shaped by the conditions of its production and circulation. As journalistic discourse is embedded in specific historical and social contexts, it is regulated by institutional norms, editorial projects, discursive practices, and material constraints (González-Arias et al., 2024). In the case of web news and short interview videos, explanations about school violence are produced within formats that prioritize immediacy, intelligibility, and relevance for broad audiences. As a result, expert knowledge is often condensed into accessible narratives that fit the conventions of these genres such as news articles, interviews and opinion pieces.

In contemporary digital media environments, the boundaries of news have become increasingly fluid, as information circulates across platforms and genres within a hybrid media system (Ekström, 2023; Ibarra Herrera, 2020). News discourse frequently consists of representations and recontextualizations of discourses originating in other settings, such as interviews, statements or expert commentaries, which are selectively reproduced for public dissemination (Ekström and Westlund, 2019). Thus, the apparent simplification of expert subjective theories in media discourse should not always be interpreted as a reduction enacted by experts themselves, but rather as the outcome of platform-specific logics and journalistic mediation. These dynamics demonstrate how subjective theories function as public interpretive frameworks, shaped by and responsive to the communicative demands of contemporary media ecosystems.

Despite its contributions, this study presents some limitations. It focuses exclusively on public discourses disseminated through media in Chile. Future research could explore other national cases, which could further illuminate how subjective theories about school violence circulate and shape beliefs and explanations about the school systems in different countries.

## 5 Implications for policy and practice

The COVID-19 pandemic was an unprecedented global experience, with no previous evidence to guide its management. It is therefore understandable that subjective theories based on personal professional knowledge and experience prevailed over explanations based on the scientific evidence that was being produced in parallel (Castro-Carrasco et al., 2024; Goller and Markert, 2024). This background is relevant to consider, given that subjective theories are beliefs whose argumentative structure justifies, but also guides the actions of professionals (Flick, 2023). If this is the case, then there is a need to advance toward the development of action plans based on the experience of schools and their stakeholders (professionals and students) during that period, in dialog with the accumulated experience of experts at the time and the scientific research produced in this regard. These plans could be integrated into universal educational policies and used by decision-makers and experts who face future situations with risks similar to those experienced in recent years (Cuadra et al., 2018), as a general reference to guide the construction of interpretations, recommendations, and action strategies (Jancic, 2015). For instance, one of the actions deemed important would be updating existing school protocols so that they respond to the needs and realities of the current generation of students.

On the other hand, this emphasis on individual responsibility could be related to possible biases that may be operating in the initial training in the disciplines of the experts considered in this study. Professional training may be prioritizing an individual perspective on the origin of the problem, paying less attention to contextual factors. Likewise, a reductionist analytical approach may be taking precedence over a broader, more systemic one that views social behavior as a message from complex mechanisms derived from different spheres. If this is the case, then it seems relevant to revisit the initial training of professionals recognized as experts in psychosocial issues (school violence, in this case), in order to include a broader understanding of how school violence develops. Likewise, it is important to raise awareness and provide training in favor of a professional disposition toward: (1) historically and institutionally situated analysis; and (2) privileging a comprehensive and interventional interdisciplinary approach. Both dimensions are aimed at advancing toward the collective and multidisciplinary construction of interpretations and proposals that ensure the relevance and effectiveness of the action strategies produced (Rindrasih et al., 2024; Tipler et al., 2018).

A general explanation of school violence from a psychological perspective centered on individuals, as it has been stated, risks being reductionist, taking the spotlight away from educational actions. However, as experts’ discourse points out, it is necessary to address the gap in socioemotional skills that increased during the pandemic period. In this respect, the role of play within the school context should not be underestimated, particularly as a means to promote collaboration and positive peer interactions, overall improving school coexistence. Non-academic activities, such as sports competitions, could contribute to strengthening students’ sense of belonging and community. Likewise, promoting creative activities that are not subject to formal assessment may encourage participation, expression, and cooperation in less pressured contexts. Thus, without necessarily opposing an explanation that emphasizes the psychological aspects of school violence toward a more contextual and educational view focused on prevention, these proposals may help reinforce a sense of agency and rise of collective self-efficacy within schools, positioning them as spaces where conflicts are addressed internally rather than displaced. For instance, to implement these proposals, the creation of multidisciplinary teams could be a practical strategy to respond to socioemotional and relational challenges in a more coordinated manner.

## 6 Conclusion

This study contributes to the understanding of expert discourse on school violence in a moment of uncertainty such as the return to face-to-face education after the pandemic. By reconstructing subjective theories disseminated in the media, it highlights how expert knowledge operates as both an interpretive framework and a media resource. The experts’ discourse describes school violence, but it also shapes public agendas, justify interventions, and allocate responsibility (Browne Monckeberg and Rodríguez-Pastene, 2019).

Studying the experts’ role in education, in a situation of social crisis such as the pandemic, provides greater understanding about the assumptions from which expert knowledge and society in general recommend proposals for addressing school violence, and recognizes the coexistence of preventive and reactive perspectives. This study has contributed to synthesizing these different conceptions, and can be useful as a comprehensive and situated framework for the design of holistic interventions and public policies that overcome reductionism and distance from the polarization between preventive or reactive approaches to school violence. As abovementioned, this can be seen in the experts’ discourse in the communications of the media.

## Data Availability

The raw data supporting the conclusions of this article will be made available by the authors, without undue reservation.
